# Synthesising Programs with Non-trivial Constants

**DOI:** 10.1007/s10817-023-09664-4

**Published:** 2023-05-13

**Authors:** Alessandro Abate, Haniel Barbosa, Clark Barrett, Cristina David, Pascal Kesseli, Daniel Kroening, Elizabeth Polgreen, Andrew Reynolds, Cesare Tinelli

**Affiliations:** 1 University of Oxford, Oxford, UK; 2 Universidade Federal de Minas Gerais, Belo Horizonte, Minas Gerais Brazil; 3 Stanford University, Stanford, USA; 4 University of Bristol, Bristol, UK; 5 Lacework Ltd, Mountain View, CA, UK; 6 Amazon Inc., Oxford, UK; 7 University of Edinburgh, Edinburgh, UK; 8 The University of Iowa, Iowa City, USA

**Keywords:** Program synthesis, Automated reasoning, Satisfiability modulo theories, Counterexample guided inductive synthesis

## Abstract

Program synthesis is the mechanised construction of software. One of the main difficulties is the efficient exploration of the very large solution space, and tools often require a user-provided syntactic restriction of the search space. While useful in general, such syntactic restrictions provide little help for the generation of programs that contain non-trivial constants, unless the user is able to provide the constants in advance. This is a fundamentally difficult task for state-of-the-art synthesisers. We propose a new approach to the synthesis of programs with non-trivial constants that combines the strengths of a counterexample-guided inductive synthesiser with those of a theory solver, exploring the solution space more efficiently without relying on user guidance. We call this approach CEGIS($\mathcal {T}$), where $\mathcal {T}$ is a first-order theory. We present two exemplars, one based on Fourier-Motzkin (FM) variable elimination and one based on first-order satisfiability. We demonstrate the practical value of CEGIS($\mathcal {T}$) by automatically synthesising programs for a set of intricate benchmarks. Additionally, we present a case study where we integrate CEGIS($\mathcal {T}$) within the mature synthesiser CVC4 and show that CEGIS($\mathcal {T}$) improves CVC4’s results.

## Introduction

Program synthesis [25] is the problem of finding a program that meets a correctness specification given as a logical formula. This is an active area of research in which substantial progress has been made in recent years.

In full generality, program synthesis is an exceptionally difficult problem, and thus, the research community has explored pragmatic restrictions. One particularly successful direction is *Syntax-Guided Program Synthesis* (SyGuS) [3]. The key idea of SyGuS is that the user supplements the logical specification with a syntactic template for the solution, defined as a context-free grammar. Leveraging the user’s intuition, SyGuS reduces the size of the solution space substantially, resulting in significant speed-ups.

Unfortunately, it is difficult to provide the syntactic template in many practical applications. A very obvious exemplar of the limits of the syntax-guided approach are programs that require non-trivial constants. In such a scenario, the syntax-guided approach requires that the user provides the exact value of the constants in the solution.

For illustration, let’s consider a user who wants to synthesise a program that rounds up a given 32-bit unsigned number *x* to the next highest power of two. If we refer to the function computed by the program as *f*(*x*), then the specification can be written as

$$ \begin{aligned} x{<}2^{31}{\Rightarrow } f(x) \& {(-f(x))}{=}f(x) \,\wedge \, f(x){\ge }x \,\wedge \, 2x{\ge }f(x) \end{aligned}$$

The first conjunct forces *f*(*x*) to be a power of two, the others require it to be the next highest. A possible solution for this is given by the following C program:
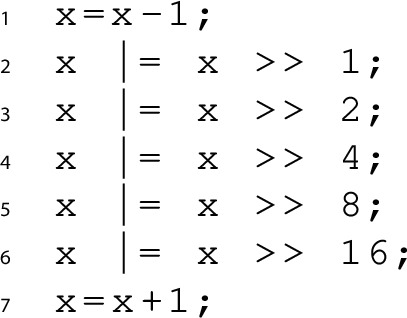


It is improbable that the user knows that the constants in the solution are exactly 1, 2, 4, 8, 16, and thus, she will be unable to effectively restrict the solution space. As a result, synthesisers are very likely to enumerate possible combinations of constants, which is highly inefficient.

In this paper we propose a new approach to the synthesis of programs with non-trivial constants that combines the strengths of a counterexample-guided inductive synthesiser with those of a solver for a first-order theory in order to perform a more efficient exploration of the solution space, without relying on user guidance. Our inspiration for this proposal is CDCL($\mathcal {T}$) [22, 31, 39] (also known as DPLL($\mathcal {T}$)), which has boosted the performance of solvers for many fragments of quantifier-free first-order logic. The CDCL($\mathcal {T}$) framework combines reasoning about the Boolean structure of a formula with reasoning about theory facts to decide satisfiability of a given first-order formula.

Similarly, we attempt to separate reasoning about a program’s structure and its constants. For this purpose, we propose a new algorithm called CounterExample-Guided Inductive Synthesis($\mathcal {T}$), where $\mathcal {T}$ is a given first-order theory for which we have a specialised solver. Analogous to its counterpart CDCL($\mathcal {T}$), the CEGIS($\mathcal {T}$) architecture features communication between a synthesiser and a theory solver, which results in a much more efficient exploration of the search space (i.e., the space of all possible programs).

While standard CEGIS architectures [27, 41] already make use of SMT solvers, the typical role of the SMT solver in existing algorithms is restricted to validating candidate solutions and providing concrete counterexamples that direct subsequent search. By contrast, CEGIS($\mathcal {T}$) allows the theory solver to communicate generalised constraints back to the synthesiser, thus enabling more significant pruning of the search space.

There are instances of more sophisticated collaboration between a program synthesiser and theory solvers. The most obvious such instance is the program synthesiser for single invocation conjectures [35] inside the CVC4 SMT solver [34]. This approach features a very tight coupling between the two components (i.e., the synthesiser and the theory solvers) that takes advantage of the particular strengths of the SMT solver by reformulating the synthesis problem as the problem of refuting a universally quantified formula (SMT solvers are better at refuting universally quantified formulae than at finding models for them). By contrast, in our approach we maintain a clear separation between the synthesiser and the theory solver while performing comprehensive and well-defined communication between the two components. This enables the flexible combination of CEGIS with a variety of theory solvers, which excel at reasoning about different kinds of constraints.

### Contributions

- We propose CEGIS($\mathcal {T}$), a program synthesis architecture that facilitates the communication between an inductive synthesiser and a solver for a first-order theory, with the objective of separating reasoning about a program’s structure and its constants.
- We present two exemplars of this architecture, one based on Fourier-Motzkin (FM) variable elimination [10] and one using an off-the-shelf SMT solver.
- As a case study, we present an integration of CEGIS($\mathcal {T}$) within the synthesiser CVC4, winner of four out of five tracks of the Syntax-Guided Synthesis (SyGuS) competition 2019. We show that CEGIS($\mathcal {T}$) improves CVC4’s performance.
- We have implemented CEGIS($\mathcal {T}$) and compared it with state-of-the-art program synthesisers on benchmarks that require intricate constants in the solution.

## Preliminaries

### The Program Synthesis Problem

Program synthesis is the task of automatically generating programs that satisfy a given logical specification. For non-recursive programs a program synthesiser can be viewed as a solver for formulae with existential second-order quantifiers.^1^

The input specification provided to a program synthesiser is of the form

1 $$\begin{aligned} \exists P .\, \forall \vec {x}.\, \sigma (P, \vec {x}) \end{aligned}$$

where *P* ranges over functions (where a function is represented by the program computing it), $\vec {x}$ is a tuple of variables ranging over the function’s inputs, and $\sigma$ is a quantifier-free formula.

### CounterExample Guided Inductive Synthesis

CounterExample-Guided Inductive Synthesis (CEGIS) is a popular approach to program synthesis. It is an iterative process that maintains at all times a candidate program $P^*$ for the specification $\exists P .\, \forall \vec {x}.\, \sigma (P, \vec {x})$. Each iteration performs inductive generalisation based on *counterexamples* provided by a verification oracle, that is, concrete input values $\vec c$ that falsify $\sigma (P^*, \vec c)$. Essentially, the inductive generalisation uses information about a limited number of inputs to make claims about all the possible inputs in the form of candidate solutions.

**Fig. 1:**
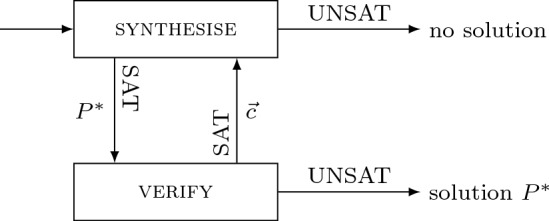
CEGIS block diagram

The CEGIS process is illustrated in Figure 1 and consists of two phases: the synthesis phase and the verification phase. Given the specification $\sigma$ of the desired program, the inductive synthesis procedure generates a candidate program $P^*$ that satisfies $\sigma (P^*,\vec {x})$ for a subset *I* of all possible inputs. The candidate program $P^*$ is passed to the verification phase, which checks whether it satisfies $\sigma (P^*, \vec {x})$ for all possible inputs. This is done by checking whether $\lnot \sigma (P^*, \vec {x})$ is unsatisfiable. If so, $\forall x.\sigma (P^*, \vec {x})$ is valid, meaning that we have successfully synthesised a solution, and the algorithm terminates. Otherwise, the verifier produces a counterexample $\vec {c}$ from the satisfying assignment for $\lnot \sigma (P^*,\vec {x})$, which is then added to the set *I* of inputs passed to the synthesiser, and the loop repeats.

The methods used in the synthesis and verification blocks vary in different CEGIS implementations. We give details of the algorithms used in CVC4 in Sect. 5.1 as an exemplar.

### CDCL($\mathcal {T}$)

To improve the performance of the traditional CEGIS process, we have devised an extension of it, CEGIS($\mathcal {T}$), inspired by the CDCL($\mathcal {T}$) framework. The latter is an extension of the CDCL algorithm used by most propositional SAT solvers [39], by a theory $\mathcal {T}$. We give a brief overview of CDCL($\mathcal {T}$) and compare CDCL($\mathcal {T}$) with CEGIS($\mathcal {T}$) next.

**Fig. 2:**
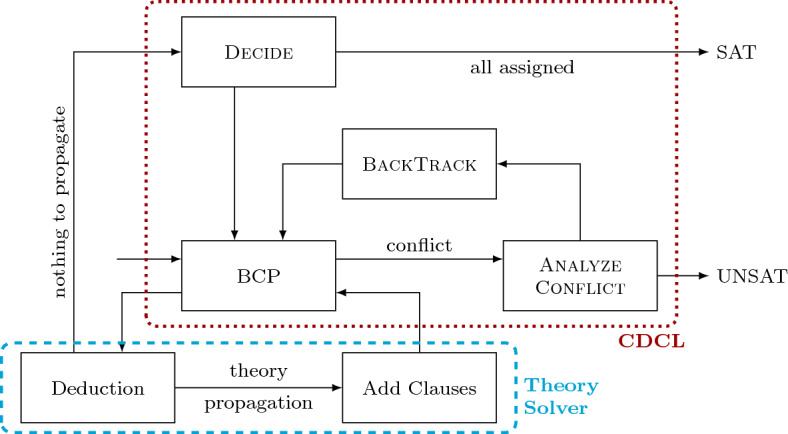
CDCL($\mathcal {T}$) with theory propagation

Given a formula *F* from a theory $\mathcal {T}$, a propositional formula $F_p$ is created from *F* in which the theory atoms are replaced by Boolean variables (the “propositional skeleton”). The standard CDCL algorithm, comprising Decide, Boolean Constraint Propagation (BCP), Analyze-Conflict and BackTrack components as illustrated in Figure 2, generates an assignment to the Boolean variables in $F_p$. The theory solver then checks whether this assignment is still consistent when the Boolean variables are replaced by their original atoms. If so, a satisfying assignment for *F* has been found. Otherwise, a constraint over the Boolean variables in $F_p$ is passed back to Decide, and the process repeats.

In the very first SMT solvers, the SAT solver first obtained a full assignment to the Boolean variables that comprise the abstraction of the input. Subsequently, the theory solver would determine whether or not this assignment was satisfiable according to the background theory. If not, the next Boolean assignment was then checked. Such SMT solvers were prone to enumerating all possible candidate solutions at the Boolean level in the worst case. The key improvement in CDCL($\mathcal {T}$) was the ability to pass back a more general constraint over the variables in the formula as a conflict clause [22], which could block not only the failed solution but a whole set of them. Furthermore, modern variants of CDCL($\mathcal {T}$) call the theory solver on partial assignments to the variables in $F_p$, which helps detect conflicts and propagations eagerly. Our proposed, new synthesis algorithm offers equivalents of both of these ideas. As we describe in Sect. 4, our implementation of CEGIS($\mathcal {T}$) may establish that the synthesis specification has no solution of a particular syntactic shape, that is, matching a particular template, regardless of the choices made to instantiate the template.

### Fourier–Motzkin Elimination

Fourier–Motzkin elimination is a mathematical algorithm for eliminating variables from a system of linear inequalities. In particular, given a system of linear inequalities of the form

$$\begin{aligned} a_1 x_1 + a_2 x_2 + \ldots + a_n x_n \le b_i, \qquad i{=}1 \ldots m \end{aligned}$$

we eliminate $x_n$ as described next. For each inequality $a_1 x_1 + a_2 x_2 + \ldots + a_n x_n \le b_i$, we get

$$\begin{aligned} x_n \le (b_i - a_1x_1 - \ldots - a_{n-1}x_{n-1})/a_n \end{aligned}$$

or

$$\begin{aligned} x_n \ge (b_i - a_1x_1 - \ldots - a_{n-1}x_{n-1})/a_n \end{aligned}$$

depending on whether $a_n > 0$ or $a_n < 0$, respectively.

This gives us a collection of upper bounds

$$\begin{aligned} x_n \le U_1(x_1, \ldots , x_{n-1}), \ldots , x_n \le U_l(x_1, \ldots , x_{n-1}) \end{aligned}$$

and lower bounds

$$\begin{aligned} x_n \ge L_1(x_1, \ldots , x_{n-1}), \ldots , x_n \ge L_p(x_1, \ldots , x_{n-1}) \end{aligned}$$.

The initial system of inequalities is equivalent to

$$\begin{aligned}{} & {} max( L_1(x_1, \ldots ,x_{n-1}), \ldots , L_p(x_1, \ldots , x_{n-1}))\\{} & {} \quad \le min(U_1(x_1, \ldots , x_{n-1}), \ldots ,U_l(x_1,\ldots , x_{n-1})) \end{aligned}$$

which is equivalent to $p \cdot l$ inequalities of the form

$$\begin{aligned} U_k(x_1, \ldots , x_{n-1}) \le L_j(x_1, \ldots , x_{n-1}) \qquad k=1\cdots l, j=1 \cdots p \end{aligned}$$.

We transformed the original system of linear inequalities into another system where $x_n$ is eliminated.

## Motivating Examples

### CEGIS on a Simple Example

In each iteration of a standard CEGIS loop, the communication from the verification phase back to the synthesis phase is restricted to concrete counterexamples. This is particularly detrimental when synthesising programs that require non-trivial constants. In such a setting, it is typical that a counterexample provided by the verification phase only eliminates a single candidate solution and, consequently, the synthesiser ends up enumerating all possible constants.

For illustration, let’s consider the trivial problem of synthesising a function *f*(*x*) where $f(x) < 0$ if $x < 334455$ and $f(x) = 0$, otherwise. One possible solution is $f(x)= ite ~ (x<334455)~ {-1}~ 0$, where $ite$ stands for *if then else*.

In order to make the synthesis task even simpler, we are going to assume that we know a part of this solution, namely we know that it must be of the form $f(x)= ite ~ (x < \textsf {?})~ {-1}~ 0$, where “$\textsf {?}$” is a placeholder for the missing constant that we must synthesise. A plausible scenario for a run of CEGIS is presented next: the synthesis phase guesses $f(x)= ite ~ (x<0)~ {-1}~ 0$, for which the verification phase returns $x=0$ as a counterexample. In the next iteration of the CEGIS loop, the synthesis phase guesses $f(x)= ite ~ (x<1)~ {-1}~ 0$ (which works for $x=0$) and the verifier produces $x=1$ as a counterexample. Following the same pattern, the synthesis phase will enumerate all the candidates

$$\begin{aligned}&f(x)= ite ~ (x<2)~ {-1}~ 0\\&\qquad \ldots \\&f(x)= ite ~ (x<334454)~{-1}~ 0 \end{aligned}$$

before finding the solution. This is caused by the fact that each of the concrete counterexamples $0,\ldots ,334454$ eliminates one candidate only from the solution space. To avoid this behavior we need to propagate more information from the verifier to the synthesis phase in each iteration of the CEGIS loop.

### Proving Properties of Programs

Synthesis engines can be used as reasoning engines in program analysers, and constants are important for this application. In such a case, the synthesised program computes the program proof of interest (e.g., program invariant [13] for safety proving, counter-model [11] for bug finding, ranking function [21] for termination proving, recurrence set [26] for non-termination). In the examples given in the rest of this section, we refer directly to the formula corresponding to each program proof computed by a synthesised program.

*Proving safety*

Let’s start by considering the very simple program below, which increments a variable *x* from 0 to 100000 and asserts that its value is less than 100005 on exit from the loop.
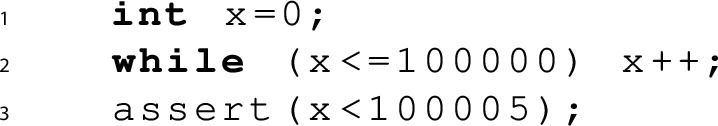


Proving the safety of such a program, i.e., that the assertion at line 3 is not violated in any execution of the program requires the generation of a loop invariant, a task well-suited for synthesis (the Syntax Guided Synthesis Competition [6] has had a track dedicated to synthesising safety invariants since 2015). For this example, a safety invariant is $x<100002$, which holds on entrance to the loop, is inductive with respect to the loop’s body, and implies the assertion on exit from the loop.

While it is very easy for a human to find this invariant, the need for a non-trivial constant makes it exceedingly difficult for state-of-the-art synthesisers: both CVC4 (version 1.5) [35] and EUSolver (version 2017-06-15) [4] fail to find a solution in an hour.

*Proving termination* Next, let’s look at the following terminating program:
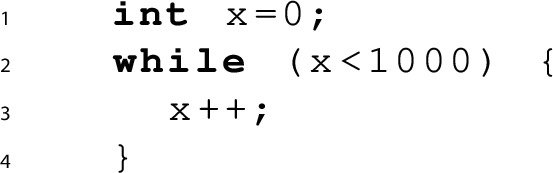


Its termination argument can be encoded as the following formula, where *R* is a *ranking function*, i.e., an injective function that has a well-founded set *D* with order $\prec$ as co-domain and is injective and monotonically decreasing with respect to the program’s transition relation:

$$\begin{aligned} \forall x.\; x{<}1000 \rightarrow 0 \prec R(x) \wedge R(x+1) \prec R(x) \end{aligned}$$

A possible ranking function is $R(x)=1000-x$ with $(D, {\prec }) = ({\mathbb {N}}, {<})$, which also requires a non-trivial constant.

*Proving non-termination* One way of proving non-termination is by finding a recurrence set, i.e., a nonempty set of states *S* such that for each state $s \in S$ there exists a transition to some $s' \in S$ [26]. As an example, let us investigate the termination behaviour of the program below:
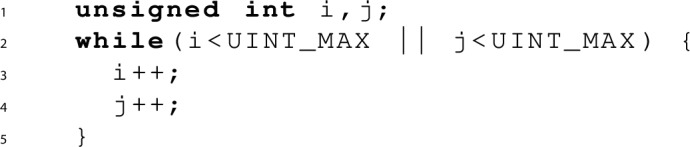


For bit-vectors, the initial state $(i_0, j_0) = (\texttt {!UINT\_MAX!},\, \texttt {!UINT\_MAX!}-1)$ leads to an infinite loop since *i* and *j* will overflow and be reset to 0 in subsequent loop iterations. The corresponding recurrence set *S* has to satisfy the following formula of bitvector arithmetic, which encodes that *S* must be reachable from an initial state and, for each state in *S*, at least one successor must be in *S*. Note that, as the program is deterministic, all successors of a state in *S* must be in *S*.

$$\begin{aligned} \exists i_0,j_0 . \forall i,j .&S(i_0,j_0) ~\wedge \\&S(i,j) \rightarrow (i{<}UINT\_MAX \vee j{<}UINT\_MAX) ~ \wedge \\&S(i,j) \rightarrow S(i+1,j+1) \end{aligned}$$

A possible recurrence set is $S(i,j)=i{<}UINT\_MAX \vee j{<}UINT\_MAX$, which again requires large constants.

*Bug finding* Next, let’s look at the buggy example below, where we increment *x* and *y* in each loop iteration, maintaining the same initial difference of 10 between them. Consequently, the assertion at line 6 fails.
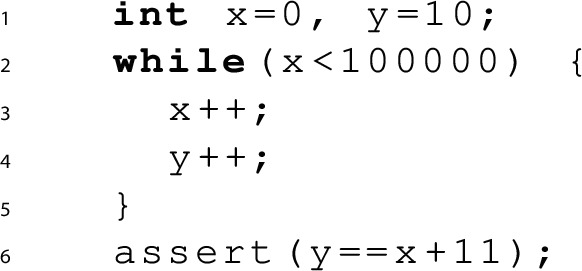


We can prove that this program has a bug by finding a *danger invariant* [14], which can be seen as a compact representation of an error trace. A danger invariant *D*(*x*, *y*) must hold in some initial state, be inductive with respect to the transition relation and, on exit from the loop, imply the negation of the assertion:

$$\begin{aligned} \exists x_0,y_0.&x_0{=}0 \wedge y_0{=}10 \wedge D(x_0,y_0) ~\wedge \\ \forall x,y .&D(x,y) \wedge x{<}100000 \rightarrow D(x+1,y+1) ~\wedge \\ \forall x,y .&D(x,y) \wedge x{\ge }100000 \rightarrow y{\ne }x{+}11 \end{aligned}$$

For our example, a possible danger invariant is $D(x,y) = (y{=}x{+}10)$. However this is not quite enough to conclude that the assertion does fail, since we have not yet established that the loop terminates from any *D*-state — thus we are in the situation where the danger invariant denotes either an assertion violation or the presence of a recurrence set.

If we want to prove only an assertion violation, an additional part of the danger invariant is a ranking function *R*(*x*, *y*) proving that the loop does terminate making the assertion at line 6 reachable.

$$\begin{aligned} \exists x_0,y_0.&x_0{=}0 \wedge y_0{=}10 \wedge D(x_0,y_0) ~\wedge \\ \forall x,y .&D(x,y) \wedge x{<}100000 \rightarrow D(x+1,y+1) \wedge R(x,y)>0 ~\wedge \\&R(x,y)>R(x+1,y+1) ~\wedge \\ \forall x,y .&D(x,y) \wedge x{\ge }100000 \rightarrow y{\ne }x{+}11 \end{aligned}$$

In this case, a ranking function is $R(x,y)=100000-x$. As we can see, both *D*(*x*, *y*) and *R*(*x*, *y*) require non-trivial constants.

## CEGIS($\mathcal {T}$)

### Overview

In this section, we describe the architecture of CEGIS($\mathcal {T}$), which is obtained by augmenting the standard CEGIS loop with a theory solver. Since we are particularly interested in the synthesis of programs with constants, we present CEGIS($\mathcal {T}$) from this particular perspective. In such a setting, CEGIS is responsible for synthesising program skeletons, whereas the theory solver generates constraints over the literals that denote constants. These constraints are then propagated back to the synthesiser.

To explain the main ideas behind CEGIS($\mathcal {T}$) in more detail, it is useful to differentiate between a candidate solution, a candidate solution skeleton, a generalised candidate solution and a finalised solution.

#### Definition 1

(*Candidate solution*) Using the notation from Sect. 2.2, a program $$P^*$$ is a *candidate solution* of $$\forall \vec x.\sigma (P,\vec x)$$ if the set $$\{ \sigma (P^*,\vec e) \mid \vec e \in E_ inputs \}$$ is satisfiable where $$E_ inputs $$ is a set of possible values for $$\vec {x}$$.

The set $$\{ \sigma (P^*,\vec e) \mid \vec e \in E_ inputs \}$$ in Definition 1 contains the ground instances of $$\sigma (P,\vec {x})$$ obtained by instantiating the vector $$\vec {x}$$ with each vector in $$E_ inputs $$. Such instances do not have any free variables other than the second-order variable *P*. If this set is satisfiable for some value (i.e., program)  for *P* then  meets the specification for each input from $$E_ inputs $$ (i.e., $$\forall ~\vec {x}\in E_ inputs .\sigma (P,\vec {x})$$).

#### Definition 2

(*Candidate solution skeleton*) Given a candidate solution $P^*$, the *skeleton* of , denoted by $P^*[\textsf {?}]$, is obtained by replacing each constant in  with a distinguished symbol $\textsf {?}$, representing a hole.

#### Definition 3

(*Generalised candidate solution*) Given a candidate solution skeleton $P^*[\textsf {?}]$, we obtain a *generalised candidate* $P^*[\vec {v}]$ by filling each hole in $P^*[\textsf {?}]$ with a distinct logical variable, i.e., variable $v_i$ will correspond to the *i*-th hole (in some arbitrary but fixed ordering of the hole occurrences in $P^*$). Then $\vec {v}=[v_1, \ldots , v_n]$, where *n* denotes the number of holes in $P[\textsf {?}]$.

#### Definition 4

(*Finalised solution*) A candidate solution $P^*$ is a *finalised solution* if the formula $\forall \vec {x}.\sigma (P^*,\vec {x})$ is valid.

#### Example 1

(Candidate solution, candidate solution skeleton, generalised candidate solution, finalised solution) Given the example in Sect. 3.1, if $E_ inputs =\{0\}$, then $f(x)=-2$ is a candidate solution. The corresponding candidate skeleton is $f[\textsf {?}](x)=\textsf {?}$ and the generalised candidate is $f[v_1](x)=v_1$. A finalised solution for this example is $f(x)= ite ~(x<334455)~ {-1}~ 0.$

**Fig. 3:**
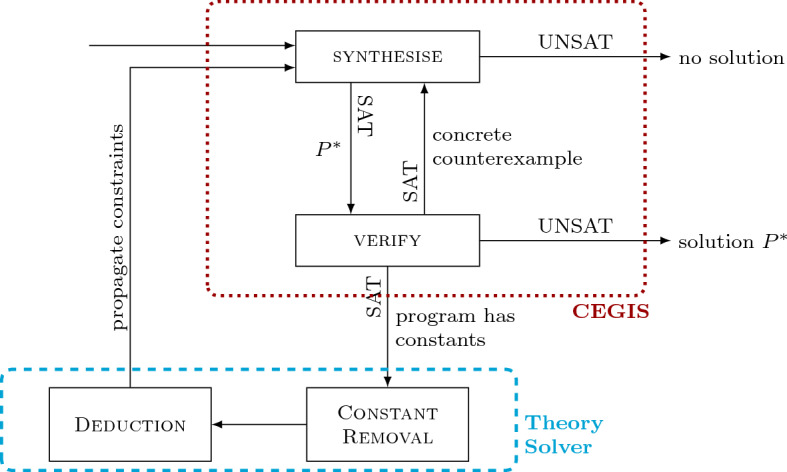
CEGIS($$\mathcal {T}$$)

Figure 3 illustrates the communication between the synthesiser and the theory solver in CEGIS($\mathcal {T}$). The interaction can be described as follows:

- The CEGIS architecture (enclosed in a dotted rectangle with the label “CEGIS”) generates the candidate solution $P^*$, which is provided to the theory solver.
- The theory solver (enclosed in a dashed rectangle with the label “Theory Solver”) obtains the skeleton $P^*[\textsf {?}]$ of $P^*$ and generalises it to $P^*[\vec {v}]$ in the box marked constant removal. Subsequently, Deduction attempts to find a constraint over $\vec {v}$ describing those values for which $P^*[\vec {v}]$ is a finalised solution.
  This constraint is propagated back to CEGIS. Whenever there is no valuation of $\vec {v}$ for which $P^*[\vec {v}]$ becomes a finalised solution, the constraint needs to block the current skeleton $P^*[\textsf {?}]$.

The CEGIS($\mathcal {T}$) algorithm is given as Algorithm 1 and proceeds as follows:

- Before entering the while loop, $E_{inputs}$ is initialized with the empty set. This means that, in the first iteration of CEGIS($\mathcal {T}$), there are no inputs to be considered and any program will trivially obey the specification.
- **CEGIS synthesis phase:** checks the satisfiability of $$\{ \sigma (P, \vec e) \mid \vec e \in E_ inputs \}$$, where $$E_ inputs $$ is a subset of all possible values for $$\vec {x}$$, and obtains a candidate solution $$P^*$$. If this set is unsatisfiable, then the synthesis problem has no solution.
- **CEGIS verification phase:** checks whether there exists a concrete counterexample for the current candidate solution $P^*$ by checking the satisfiability of the formula $\lnot \sigma (P^*, \vec {x})$. If the result is UNSAT, then $P^*$ is a finalised solution to the synthesis problem. If the result is SAT, a concrete counterexample $\vec c$ can be extracted from the satisfying assignment.
- **Theory solver:** if $P^*$ contains constants, then they are eliminated, resulting in the skeleton $P^*[\textsf {?}]$, which is afterwards generalised to $P^*[\vec {v}]$. The goal of the theory solver is to find $\mathcal {T}$-implied literals and communicate them back to the CEGIS part in the form of a constraint, $C(P, P^*, \vec {v})$. In Algorithm 1, this is done by $Deduction (\sigma , P^*[\vec {v}])$. The result of $Deduction (\sigma , P^*[\vec {v}])$ is of the following form: whenever there exists a valuation of $\vec {v}$ for which the current skeleton $P^*[\textsf {?}]$ is a finalised solution, $res {=} true$ and $C(P, P^*, \vec {v}){=}\bigwedge _{i=1{\cdot }n} v_i{=}c_i$, where $c_i$ are constants; otherwise, $res {=} false$ and $C(P, P^*, \vec {v})$ needs to block the current skeleton $P^*[\textsf {?}]$, i.e., $C(P, P^*, \vec {v}){=}P[\textsf {?}]{\ne }P^*[\textsf {?}]$. In our CEGIS implementation, this amounts to placing constraints over the boolean selector variables in the synthesis formula, which choose the sequence of operators and operands in the candidate program $P^*$.
- **CEGIS learning phase:** adds new information to the problem specification. If we did not use the theory solver (i.e., the candidate $$P^*$$ found by the synthesiser did not contain constants or the problem specification was out of the theory solver’s scope), then the learning would be limited to adding the concrete counterexample $$\vec e$$ obtained from the verification phase to the set $$E_ inputs $$. However, if the theory solver is used and returns $$ res {=} true $$, then the second element in the tuple contains valuations for $$\vec {v}$$ such that $$P^*[\vec {v}]$$ is a finalised solution. If $$ res {=} false $$, then the second element blocks the current skeleton and needs to be added to $$\sigma $$.

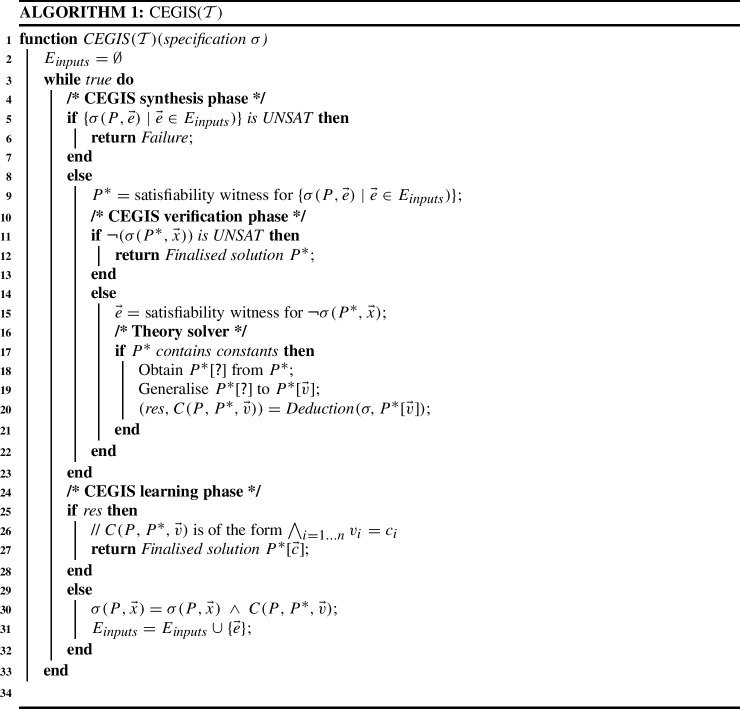


### CEGIS($\mathcal {T}$) with a Theory Solver Based on FM Elimination

In this section we describe a theory solver based on FM variable elimination. In our case, we call the FM theory solver whenever the specification $\sigma$ belongs to linear arithmetic. Otherwise, the FM theory solver is not called.

As mentioned above, we need to produce a constraint over variables $\vec {v}$ describing the situation when $P^*[\vec {v}]$ is a finalised solution. For this purpose, we consider the formula $\exists \vec {x}.\, \lnot \sigma (P^*[\vec {v}], \vec {x}),$ where $\vec {v}$ is a satisfiability witness if the specification $\sigma$ admits a counterexample $\vec {x}$ for $P^*$. Let $E(\vec {v})$ be the formula obtained by eliminating $\vec {x}$ from $\exists \vec {x}.\, \lnot \sigma (P^*[\vec {v}], \vec {x})$. If $\lnot E(\vec {v})$ is satisfiable, any satisfiability witness gives us the necessary valuation for $\vec {v}$:

$$\begin{aligned} C(P, P^*, \vec {v})=\bigwedge _{i=1{\cdot }n} v_i=c_i\ . \end{aligned}$$

If $\lnot E(\vec {v})$ is UNSAT, then the current skeleton $P^*[\textsf {?}]$ needs to be blocked. This reasoning is supported by Lemma 1 and Corollary 1.

#### Lemma 1

Let $E(\vec {v})$ be the formula obtained by eliminating $\vec {x}$ from $\exists \vec {x}.\, \lnot \sigma (P^*[\vec {v}],\vec {x})$. Then, any witness $\vec {v^{\#}}$ to the satisfiability of $\lnot E(\vec {v})$ gives us a finalised solution $P^*[\vec {v^{\#}}]$ to the synthesis problem.

#### Proof

From the fact that $E(\vec {v})$ is obtained by eliminating $\vec {x}$ from $\exists \vec {x}.\, \lnot \sigma (P^*[\vec {v}], \vec {x})$, we get that $E(\vec {v})$ is equivalent with $\exists \vec {x}.\, \lnot \sigma (P^*[\vec {v}], \vec {x})$ (we use $\equiv$ to denote equivalence):

$$\begin{aligned} E(\vec {v}) \equiv \exists \vec {x}.\, \lnot \sigma (P^*[\vec {v}], \vec {x})\ . \end{aligned}$$

Then:

$$\begin{aligned} \lnot E(\vec {v}) \equiv \forall \vec {x}.\, \sigma (P^*[\vec {v}], \vec {x})\ . \end{aligned}$$

Consequently, any $\vec {v^{\#}}$ satisfying $\lnot E(\vec {v})$ also satisfies $\forall \vec {x}.\, \sigma (P^*[\vec {v}], \vec {x})$. From $\forall \vec {x}.\, \sigma (P^*[\vec {v^{\#}}], \vec {x})$ and Definition 4 we get that $P^*[\vec {v^{\#}}]$ is a finalised solution.

$$\square $$

#### Corollary 1

Let *E*(*v*) be the formula that is obtained by eliminating $\vec {x}$ from $\exists \vec {x}.\, \lnot \sigma (P^*[\vec {v}], \vec {x})$. If $\lnot E(\vec {v})$ is unsatisfiable, then the corresponding synthesis problem does not admit a solution for the skeleton $P^*[\textsf {?}]$.

#### Proof

Given that $\lnot E(\vec {v}) \equiv \forall \vec {x}.\, \sigma (P^*[\vec {v}], \vec {x})$, if $\lnot E(\vec {v})$ is unsatisfiable, so is $\forall \vec {x}.\, \sigma (P^*[\vec {v}], \vec {x})$, meaning that there is no valuation for $\vec {v}$ such that the specification $\sigma$ is obeyed for all inputs $\vec {x}$. $\square$

For the current skeleton $P^*[\textsf {?}]$, the constraint $E(\vec {v})$ generalises the concrete counterexample $\vec e$ (found during the CEGIS verification phase) in the sense that the instantiation $\vec {v}^{\#}$ of $\vec {v}$ for which $\vec e$ failed the specification, i.e., $\lnot \sigma (P^*[\vec {v}^{\#}], \vec e)$, is a satisfiability witness for $E(\vec {v})$. This is true as $E(\vec {v}) \equiv \exists \vec {x}.\, \lnot \sigma (P^*[\vec {v}], \vec {x})$, which means that the satisfiability witness $(\vec {v}^{\#},\vec e)$ for $\lnot \sigma (P^*[\vec {v}], \vec {x})$ projected on $\vec {v}$ is a satisfiability witness for $E(\vec {v})$.

#### Disjunction

The specification $\sigma$ and the candidate solution may contain disjunctions. However, most theory solvers (and in particular the FM variable elimination [10]) work on conjunctive fragments only. A naïve approach could use case-splitting, i.e., transforming the formula into Disjunctive Normal Form (DNF) and then solving each clause separately. This can result in a number of clauses exponential in the size of the original formula. Instead, we handle disjunction using the Boolean Fourier-Motzkin procedure [28, 43]. As a result, the constraints we generate may be non-clausal.

#### Applying CEGIS($\mathcal {T}$) with FM to the Motivational Example

We recall the example in Sect. 3 and apply CEGIS($\mathcal {T}$). The problem is

$$\begin{aligned} \exists f. \forall x.\, x< 334455 \rightarrow f(x) < 0 \wedge x{\ge }334455 \rightarrow f(x) = 0 \end{aligned}$$

which gives us the following specification:

$$\begin{aligned} \sigma (f,x) = (x \ge 334455 \vee f(x)< 0) \wedge (x{<}334455 \vee f(x) = 0)\ . \end{aligned}$$

The first synthesis phase generates the candidate $f^*(x){=}0$ for which the verification phase returns the concrete counterexample $x{=}0$. As this candidate contains the constant 0, we generalise it to $f^*[v_1](x) = v_1$, for which we get

$$\begin{aligned} \sigma (f^*[v_1],x)=(x \ge 334455 \vee v_1< 0) \wedge (x{<}334455 \vee v_1 = 0)\ . \end{aligned}$$

Next, we use FM to eliminate *x* from $f^*$:

$$\begin{aligned} \exists x.\lnot (\sigma (f^*[v_1],x))=\exists x. (x < 334455 \wedge v_1 \ge 0) \vee (x{\ge }334455 \wedge v_1 \ne 0)\ . \end{aligned}$$

Note that, given that formula $\lnot \sigma (f^*[v_1],x)$ is in DNF, for convenience we directly apply FM to each disjunct and obtain $E(v_1)=v_1{\ge }0 \vee v_1{\ne }0$, which characterises all the values of $v_1$ for which there exists a counterexample. When negating $E(v_1)$ we get $v_1{<}0 \,\wedge \, v_1{=}0$, which is UNSAT. As there is no valuation of $v_1$ for which the current $f^*$ is a finalised solution, the result returned by the theory solver is $( false , f[\textsf {?}]{\ne }f^*[\textsf {?}])$, which is used to augment the specification. Subsequently, a new CEGIS($\mathcal {T}$) iteration starts. The learning phase has changed the specification $\sigma$ to

$$\begin{aligned} \sigma (f,x) = (x{\ge }334455 \vee f(x){<}0) \wedge (x{<}334455 \vee f(x){=}0) \wedge f[\textsf {?}]{\ne }\textsf {?}\ . \end{aligned}$$

This forces the synthesis phase to pick a new candidate solution with a different skeleton. The new candidate solution we get is $f^*(x)= ite ~(x{<}100) ~-3 ~1$, which works for the previous counterexample $x{=}0$. However, the verification phase returns the counterexample $x{=}100$. Again, this candidate contains constants which we replace by symbolic variables, obtaining

$$\begin{aligned} f^*[v_1,v_2,v_3](x)= ite ~(x{<}v_1)~ v_2 ~v_3\ . \end{aligned}$$

Next, we use FM to eliminate *x* from

$$\begin{aligned}&\exists x.\lnot (\sigma (f^*[v_1,v_2,v_3],x))=\\&\exists x. \lnot (x{\ge }334455 \vee (x{<}v_1 \rightarrow v_2{<}0 \wedge x{\ge }v_1\rightarrow v_3{<}0) \wedge \\&\quad x{<}334455 \vee (x{<}v_1 \rightarrow v_2{=}0 \wedge x{\ge }v_1\rightarrow v_3{=}0))=\\&\exists x.\lnot ((x{\ge }334455 \vee x{\ge }v_1 \vee v_2{<}0) \wedge (x{\ge }334455 \vee x{<}v_1 \vee v_3{<}0) \wedge \\&\quad (x{<}334455 \vee x{\ge }v_1 \vee v_2{=}0) \wedge (x{<}334455 \vee x{<}v_1 \vee v_3{=}0))=\\&\exists x.(x{<}334455 \wedge x{<}v_1 \wedge v_2{\ge }0) \vee (x{<}334455 \wedge x{\ge }v_1 \wedge v_3{\ge }0) \vee \\&\quad (x{\ge }334455 \wedge x{<}v_1 \wedge v_2{\ne }0) \vee (x{\ge }334455 \wedge x{\ge }v_1 \wedge v_3{\ne }0)\ . \end{aligned}$$

As we work with integers, we can rewrite $x{<}334455$ to $x{\le }334454$ and $x{<}v_1$ to $x{\le }v_1{-}1$. Then, we obtain the following constraint $E(v_1,v_2,v_3)$ (as aforementioned, we applied FM to each disjunct in $\lnot \sigma (f^*[v_1,v_2,v_3],x))$

$$\begin{aligned} E(v_1,v_2,v_3)=v_2{\ge }0 \vee (v_1{\le }334454 \wedge v_3{\ge }0) \vee (v_1{\ge }334456 \wedge v_2{\ne }0) \vee v_3{\ne }0 \end{aligned}$$

whose negation is

$$\begin{aligned} \lnot E(v_1,v_2,v_3)=v_2{<}0 \wedge (v_1{>}334454 \vee v_3{<}0) \wedge (v_1{<}334456 \vee v_2{=}0) \wedge v_3{=}0 \end{aligned}$$

A satisfiability witness is $v_1{=}334455$, $v_2{=}-1$ and $v_3{=}0$. Thus, the result returned by the theory solver is $( true ,v_1{=}334455 \wedge v_2{=}-1 \wedge v_3{=}0)$, which is used by CEGIS to obtain the finalised solution

$$\begin{aligned} f^*(x)= ite ~(x{<}334455)~ {-1}~ 0 \ . \end{aligned}$$

### CEGIS($\mathcal {T}$) with an SMT-Based Theory Solver

For our second variant of a theory solver, we make use of an off-the-shelf SMT solver that can solve first-order formulae with quantifiers. This approach is more general than the one described in Sect. 4.2, as there are solvers for a broad range of theories.

Recall that our goal is to obtain a constraint $C(P,P^*,\vec {v})$ that either characterises the valuations of $\vec {v}$ for which $P^*[\vec {v}]$ is a finalised solution or blocks $P^*[\textsf {?}]$ whenever no such valuation exists. Consequently, we use the SMT solver to check the satisfiability of the formula

$$\begin{aligned} \varPhi =\exists \vec {v} \,\forall \vec {x}.\, \sigma (P^*[\vec {v}], \vec {x})\ . \end{aligned}$$

If $\varPhi$ is satisfiable, then any satisfiability witness $\vec {c}$ gives us a valuation for $\vec {v}$ such that $P^*$ is a finalised solution: $C(P,P^*, \vec {v}) = \bigwedge _{i=1{\cdot }n} v_i=c_i.$ Conversely, if $\varPhi$ is unsatisfiable, then $C(P,P^*, \vec {v})$ must block the current skeleton $P^*[\textsf {?}]$: $C(P,P^*, \vec {v}) = P[\textsf {?}]\ne P^*[\textsf {?}].$

The formula passed to the SMT solver is still a non-trivial formula containing an alternating quantifier, except now both quantifier prefixes are first-order, that is quantify over domain values not over functions. It would not be uncommon for an SMT solver to take substantially longer to solve this formula than the synthesis step of CEGIS takes. To avoid that, we impose a heuristically chosen timeout of 2 s on the verification step. If the solver exceeds the timeout, CEGIS($\mathcal {T}$) defaults to the behaviour of a standard CEGIS loop for the current iteration, and returns the concrete counterexample found by the CEGIS verification phase (i.e., the satisfiability witness for $\lnot \sigma (P^*,\vec {x})$).

To reduce the number of timeouts, we produce several formulae, each of which constrains $\varPhi$ in a different way. These formulae are then passed to the SMT solver sequentially with a timeout. For each variable *v* in $\vec {v}$, we produce two formulae: the first, $\varPhi \wedge v{<}K$, constrains *v* to be smaller than the value *K* it took in the original candidate program found by the CEGIS synthesis phase (i.e., its corresponding value in $P^*$ found by the CEGIS synthesis phase); the second, $\varPhi \wedge v{>}K$, constrains it to be greater than *K*. Table 1 captures the possible outcomes in terms of the resulting $C(P,P^*,\vec {v})$. In column 2, both $\varPhi \wedge v{<}K$ and $\varPhi \wedge v{>}K$ are unsatisfiable, meaning that the current program skeleton $P^*[\textsf {?}]$ is blocked. In column 3 and 4, only one of the formulae is proved to be unsatisfiable, meaning that the skeleton is only blocked for the corresponding subdomain of *v*. If both SMT calls time out, as captured by column 5, then, for the current iteration, CEGIS($\mathcal {T}$) defaults to the behavior of a standard CEGIS loop and returns the concrete counterexample found by the CEGIS verification phase. The last two columns capture the scenario where one of the SMT calls returns with a valuation for *v*.

**Table 1:** Learned constraints for each SMT result combination (✗: unsat, $\checkmark$: sat, $\emptyset$: timeout, $*$: any result)

$\varPhi \wedge v<K$	✗	✗	$\emptyset$	$\emptyset$	$\checkmark$	$*$
$\varPhi \wedge v>K$	✗	$\emptyset$	✗	$\emptyset$	$*$	$\checkmark$
$C(P,P^*,\vec {v})$	$P[\textsf {?}]{\ne } P^*[\textsf {?}]$	$P[\textsf {?}]{\ne } P^*[\textsf {?}] \wedge v{<}K$	$P[\textsf {?}]{\ne } P^*[\textsf {?}] \wedge v{>}K$	*K*	$v{=}c$	$v{=}c$

#### Applying SMT-Based CEGIS($\mathcal {T}$) to the Motivational Example

Again, we recall the example in Sect. 3. We will solve it by using SMT-based CEGIS($\mathcal {T}$) for the theory of linear arithmetic. For this purpose, we assume that the synthesis phase finds the same sequence of candidate solutions as in Sect. 3. Namely, the first candidate is $f^*(x){=}0$, which gets generalised to $f^*[v_1](x){=}v_1$. Then, we invoke SMT twice for $\varPhi _{v_1<}$ and $\varPhi _{v_1>}$, where

$$\begin{aligned} \varPhi _{v_1<}&= \forall x. (x{\ge }334455 \vee v_1{<}0) \wedge (x{<}334455 \vee v_1{=}0) \wedge v_1 {<} 0 \ \;\\ \varPhi _{v_1>}&= \forall x. (x{\ge }334455 \vee v_1{<}0) \wedge (x{<}334455 \vee v_1{=}0) \wedge v_1 {>} 0\ . \end{aligned}$$

The SMT solver returns UNSAT for both, which means that $C(f,f^*,v_1)=f[\textsf {?}]{\ne }\textsf {?}$. The second candidate is $f^*(x)= ite ~(x<100) ~-3 ~1$, which generalises to $f^*[v_1,v_2,v_3](x)= ite ~(x<v_1)~ v_2 ~v_3.$ The corresponding base constraint for the SMT solver is $\forall x.\, \sigma (( ite ~(x<v_1)~ v_2 ~v_3), x)$, for which one SMT invocation obtains the satisfiability witness $v_1=334455$, $v_2=-1$ and $v_3=0$. Then $C(f, f^*, v_1,v_2,v_3)=v_1{=}334455 \wedge v_2{=}-1 \wedge v_3{=}0$, which gives us the same finalised solution we obtained when using FM in Sect. 3.

## Case Study: CEGIS($\mathcal {T}$) Within CVC4

In this section, we discuss the use of CEGIS($\mathcal {T}$) to improve the search of the solution space of an existing and mature synthesiser, CVC4, winner of four out of five tracks of the Syntax-Guided Synthesis (SyGuS) competition 2019 [44]. We start by giving a general description of the internals of CVC4, followed by discussing the actual embedding of CEGIS($\mathcal {T}$). For an in-depth description see [34].

### Enumerative Synthesis in CVC4

CVC4 makes use of several strategies for solving synthesis problems [35]. In this paper, we focus on its enumeration-based techniques [34]. We have integrated two variants of CEGIS($\mathcal {T}$) as extensions of these techniques. Both are based on incorporating a theory solver for finding values of holes in candidate solutions. Before describing these extensions, we introduce necessary background details of how term enumeration is encoded inside an SMT solver.

*Term Enumeration via Deep Embedding* CVC4 uses a specialised technique for enumeration-based synthesis that is based on encoding a given SyGuS grammar as an algebraic data type [37]. Each value of this datatype can be understood as the Abstract Syntax Tree (AST) of a program generated by the grammar, as illustrated by the following example.

#### Example 2

Given a program represented as a function $P : (x:\textsf{Int}) \times (y:\textsf{Int}) \rightarrow \textsf{Int}$ and the context-free grammar *R* below, specifying which integer ($I$) and Boolean ($B$) terms can appear in candidate solutions for *P*:

$$\begin{aligned}{} & {} I{::}{=} 0 \ \mid \ 1 \ \mid \ x \ \mid \ y \ \mid \ I+ I\ \mid \ I- I\ \mid \ \textsf{ite}( B, I, I)\\{} & {} B{::}{=} B\ge B\ \mid \ I\simeq I\ \mid \ \lnot B\ \mid \ B\wedge B\end{aligned}$$

CVC4 generates the following mutually recursive datatypes:

$$\begin{aligned} \mathcal {I}= & {} \textsf{0} \ \mid \ \textsf{1} \ \mid \ \textsf{x} \ \mid \ \textsf{y} \ \mid \ \textsf{plus}( \mathcal {I}, \mathcal {I}) \ \mid \ \textsf{minus}( \mathcal {I}, \mathcal {I}) \ \mid \ \textsf{ite}( \mathcal {B}, \mathcal {I}, \mathcal {I}) \\ \mathcal {B}= & {} \textsf{geq}( \mathcal {I}, \mathcal {I}) \ \mid \ \textsf{eq}( \mathcal {I}, \mathcal {I}) \ \mid \ \textsf{not}( \mathcal {B}) \ \mid \ \textsf{and}( \mathcal {B}, \mathcal {B}) \end{aligned}$$

Each datatype constructor corresponds to a production rule of *R*, e.g., $\textsf{plus}$ corresponds to the rule $I{::}{=} I+ I$. A datatype term such as $\textsf{plus}( \textsf{x}, \textsf{y} )$ represents the arithmetic term $x+y$.

In the context of SyGuS, the given grammar captures the user-provided syntax restrictions. In the absence of such restrictions, CVC4 generates a default grammar that, roughly speaking, generates all possible programs of the specified type — for instance of type $\textsf{Int}\times \textsf{Int}\rightarrow \textsf{Int}$ for the programs in the example above. Since the aim of CEGIS($\mathcal {T}$) is to perform efficient search space exploration without relying on syntax restrictions (see Sect. 1), CVC4 with CEGIS($\mathcal {T}$) uses a default grammar in a similar way.

The correspondence between the ASTs and their denotational semantics is achieved by a deep embedding, constructed automatically by CVC4, of the new algebraic data type into CVC4’s (combined) background theory. Concretely, CVC4’s background theory is extended with *evaluation operators* $e_D$, for each generated datatype *D*, whose semantics is to interpret the datatype values of sort *D* in the theory of the type represented by *D*. For instance, $e_{\mathcal {I}}$, which takes as input a value of type $\mathcal {I}$ and two integer values for the variables *x* and *y*, respectively, is defined axiomatically so that $e_{\mathcal {I}}(\textsf{plus}(\textsf{x},\textsf{y}), 3, 4) \equiv 7$. This way, determining whether a datatype value denotes an actual solution to the synthesis problem (1) amounts to checking the satisfiability of

2 $$\begin{aligned} \forall z.\, \exists \vec {x}.\, \lnot \sigma (e_{\textsf{S}}(z,\vec {x}), \vec {x}) \end{aligned}$$

in which *z* has type *D*. If CVC4 can find datatype value , encoding a program $P^*$, such as the instance  of (2) is unsatisfiable, then $P^*$ is a solution for (1).

Depending on the structure of the synthesis problem, CVC4 uses different strategies for generating candidates solutions. It either applies a constraint-based (*smart*) enumeration, which allows for numerous optimisations [34, Section 2]; a highly optimised brute-force (*fast*) enumeration [34, Section 3]; or a *hybrid* approach combining smart and fast enumeration [34, Section 4]. Note that the integration of CEGIS($\mathcal {T}$) in CVC4 is agnostic to the enumeration strategy.

### CEGIS($\mathcal {T}$) in CVC4 via Skeleton Generation

In this section, we discuss our first approach for integrating CEGIS($\mathcal {T}$) with CVC4. This direction follows Algorithm 1 very closely. Given a candidate solution $P^*$, if the verification fails and $P^*$ contains constants, then a skeleton $P^*[?]$ is generated that replaces the constants with symbolic holes. Similar to Sect. 4.3, an off-the-shelf SMT solver, in this case CVC4 itself, which can perform efficient quantifier reasoning in the theories of linear arithmetic and bitvectors [30, 36], checks the satisfiability of

4 $$\begin{aligned} \varPhi =\exists \vec {v} \,\forall \vec {x}.\, \sigma (P^*[\vec {v}], \vec {x}). \end{aligned}$$

If $\varPhi$ is satisfiable, learning the constraint

5 $$\begin{aligned} C(P, P^*, \vec {v})=\bigwedge _{i=1{\cdot }n} v_i=c_i \end{aligned}$$

provides a solution to the original conjecture. However, differently from Algorithm 1, if $\varPhi$ is unsatisfiable, meaning that the skeleton $P^*[?]$ is infeasible for every constant value tuple $\vec {c}$, we have no way of blocking CVC4 from generating new candidates differing from $P^*$ only by the constant values. This is because our enumeration, in this first approach, is not changed so that it can reason about skeletons. That is the motivation for our second approach, described in Sect. 5.3. Instead of enumerating concrete programs, it directly enumerates skeletons, which are turned into concrete solutions by the theory solvers. However, this first approach has the advantage that it can be used with other approaches to CEGIS such as, for instance, divide and conquer [7, 8]. In that approach, the candidate solutions are built from enumerated partial solutions according to how they behave on the current set of counterexamples, which requires the enumeration to provide concrete programs rather than skeletons.

Observe that when generating $P^*[?]$ from $P^*$ not all constants can be abstracted away, since this can, for instance, transform linear problems into non-linear ones.

#### Example 3

Suppose that a candidate $P^*=1 + 2\times x$ is generated for a linear function. Abstracting the constants, we obtain $P^*[?]=v_1 + v_2\times x$, which is a non-linear query. CVC4 does not have efficient support for the combination of non-linear arithmetic and quantifiers. In such cases, the generalisation is only partial. In this case, only the first constant is abstracted, with the final skeleton being $P^*[?]=v_1 + 2\times x$.

Note that this issue only impacts conjectures with the specific pattern of having constants multiplying variables. This does not prevent the handling of conjectures with multiple constants.

### CEGIS($\mathcal {T}$) in CVC4 via Any Constant Constructors

In the second approach for CEGIS($\mathcal {T}$), we explicitly model the holes in candidate solutions using a specialised datatype constructor $?:\textsf{Int}\rightarrow \mathcal {I}$ within the datatype that encodes the syntactic restrictions of the input. We refer to this as the *any constant constructor*. Internally, our solver treats an application of this constructor as the representation of any constant of integer type. Using this constructor, the process for generating candidate skeletons is made explicit at the level of the datatype, with datatype values now representing arithmetic terms with holes. Thus, in this approach, we remove all other constructors corresponding to concrete constants from the datatypes encoding the grammar and include only this constructor instead.

#### Example 4

Consider the grammar *R* from Example 2. Using the any constant constructor, the datatype encoding of *R* becomes:

$$\begin{aligned} \mathcal {I}= & {} ?(\textsf{Int}) \ \mid \ \textsf{x} \ \mid \ \textsf{y} \ \mid \ \textsf{plus}( \mathcal {I}, \mathcal {I}) \ \mid \ \textsf{minus}( \mathcal {I}, \mathcal {I}) \ \mid \ \textsf{ite}( \mathcal {B}, \mathcal {I}, \mathcal {I})\\ \mathcal {B}= & {} \textsf{geq}( \mathcal {I}, \mathcal {I}) \ \mid \ \textsf{eq}( \mathcal {I}, \mathcal {I}) \ \mid \ \textsf{not}( \mathcal {B}) \ \mid \ \textsf{and}( \mathcal {B}, \mathcal {B}) \end{aligned}$$

Notice that the argument of the any constant constructor ? is the builtin integer type $\textsf{Int}$. This is in contrast to the grammar from Sect. 2, which had no subfields of integer type. Hence, the arithmetic theory solver of CVC4 will reason about values of this datatype in the (combined) theory of datatypes and integer arithmetic.

In this approach, CVC4 generates candidate solutions $P^*$ that can be abstracted to a skeleton $P^*[?]$ by replacing each application of an any constant constructor with a hole. For example, $\textsf{plus}( \textsf{x}, ?(i))$ becomes $x+v_1$ for fresh variable $v_1$ and some (for now irrelevant) integer value *i*. For each such skeleton, CVC4 proceeds to determine the satisfiability of $\varPhi =\exists \vec {v} \,\forall \vec {x}.\, \sigma (P^*[\vec {v}], \vec {x})$. If $\varPhi$ is unsatisfiable, the constraintis learned, which will prevent the generation of all programs differing from $P^*$ only on the constants, effectively blocking the skeleton $P^*[?]$.

An advantage of this approach with respect to the approach in the previous section is that it allows the term enumerator to reason about the equivalence of expressions with holes in order to eliminate redundant candidates, with the effect of accelerating the search. In detail, the term enumeration techniques in CVC4 employ aggressive strategies for blocking candidate solutions that are equivalent to previously generated ones. This is a critical aspect of the efficiency of an enumerative-based synthesis solver as it allows it to recognise and immediately discard redundant candidate solutions. We call this process *blocking via theory rewriting* [34] since it uses the SMT solver’s own term simplifier as an incomplete, but fast, checker for term equivalence. The process needs special consideration when extending the solver to CEGIS($\mathcal {T}$).

For example, consider the candidate solution $x+0$, whose datatype encoding is $\textsf{plus}(\textsf{x},0)$. In CVC4’s default implementation of CEGIS, the term enumerator will skip this candidate (as well as all candidates that have it as a subterm) because it is redundant with the smaller candidate $\textsf{x}$. This is a problem for the approach in Sect. 5.2, which may then miss the candidate $\textsf{plus}(\textsf{x},0)$ and and hence fail to generalise it to a finalised solution when a solution of the form $\textsf{plus}(\textsf{x},c)$ exists. By contrast, the approach in this section can be instrumented in this setting to reason about constants symbolically. In particular, instead of reasoning about the equivalence of candidate terms of the form $P^*=\textsf{plus}(\textsf{x},?(c))$ based on fixing a value for *c*, our extensions to blocking via theory rewriting with CEGIS($\mathcal {T}$) employ stronger criteria for term redundancy based on analyzing the entire set of skeleton instances. As an example, the skeleton term $\textsf{plus}(?(c1),?(c2))$ is redundant according to our criteria since the addition of two constants is always equivalent to some constant. Thus, this skeleton is considered redundant with respect to the simpler skeleton ?(*c*).

## Experimental Evaluation

We compared CEGIS($\mathcal {T}$) against CEGIS in two program synthesisers and evaluated the improvement in performance. The first synthesiser is our prototype implementation fastsynth and the second one is CVC4, reflecting the case study in Sect. 5. Both fastsynth^2^ and the implementation inside CVC4^3^ are available to download.

We conducted the experimental evaluation on an AWS c5.18xlarge^4^ We used the Linux *time* command to measure CPU time used for each benchmark. The runtime was limited to 1800 s per benchmark. We used MiniSat [17] as the SAT solver, and Z3 v4.5.1 [16] as the SMT-solver in CEGIS($\mathcal {T}$) with SMT-based theory solver. The SAT solver could, in principle, be replaced with Z3 to solve benchmarks over a broader range of theories.

### CEGIS($\mathcal {T}$) in fastsynth

The basic CEGIS implementation in fastsynth uses SAT/SMT solving for both synthesis and verification. For synthesis, we construct a formula which encodes all possible programs up to a set program length and is satisfied if one such program satisfies the logical specification. The formula introduces extra boolean “selector” variables, which choose the sequence of operators and operands in the candidate program $P^*$.

*Incremental Satisfiability Solving* The CEGIS implementation may sometimes perform hundreds of loop iterations before finding the correct solution. Recall that the synthesis block of CEGIS is based on SAT solving. Each iteration of CEGIS, the synthesis phase makes a call to a SAT solver. Consequently, we may end up making hundreds of calls to this SAT solver, which are all very similar (the same base specification with some extra constraints added in each iteration). This makes CEGIS a prime candidate for incremental SAT solving. We implemented incremental solving in the synthesis block of CEGIS.

*Benchmarks* We selected a set of bitvector benchmarks from the Syntax-Guided Synthesis (SyGuS) competition [5] and a set of benchmarks synthesising safety invariants and danger invariants for C programs [14]. The selection criterion was that the solution to be synthesized requires constants. All benchmarks are written in SyGuS-IF [33], a variant of the SMT-LIB 2 language [9].

Since the syntactic restrictions (called the *grammar* or the *template*) provided in the SyGuS benchmarks generally contain all the necessary non-trivial constants, we completely removed the grammars from these benchmarks. Removing just the non-trivial constants and keeping the rest of the grammar (with the only constants being 0 and 1) would have made the problem much more difficult, as the constants would have had to be incrementally constructed by applying the operators available to 0 and 1.

We group the 83 benchmarks into three categories: 47 fall into invariant generation, which covers danger invariants, safety invariants and the class of invariant generation benchmarks from the SyGuS competition; 6 derive from hackers/crypto, which includes benchmarks from hackers-delight and cryptographic circuits; and 7 benchmarks are categorised as comparisons, i.e., benchmarks that require synthesising longer programs with comparisons, e.g., finding the maximum value of 10 variables. The remaining 23 benchmarks are listed under other, and are all benchmarks taken from the SyGuS competition that do not fit neatly into any of the previous categories.

*Results* In Table 2 we report results comparing four different configurations of CEGIS and CEGIS($\mathcal {T}$) in fastsynth, along with a “virtual best solver” result, which is the fastest result of all the configurations. These results are from the prototype implementation of CEGIS($\mathcal {T}$) in [1]. The configurations presented in the table are as follows:

- CEGIS($\mathcal {T}$)-FM: CEGIS($\mathcal {T}$) with Fourier-Motzkin as the theory solver;
- CEGIS($\mathcal {T}$)-SMT: CEGIS($\mathcal {T}$) with Z3 as the theory solver;
- CEGIS: basic CEGIS as described in Sect. 2.2;
- CEGIS-Inc: basic CEGIS with incremental SAT solving;
- CEGIS($\mathcal {T}$)-vbs: virtual best solver. The fastest result of the above four configurations.

The results for our implementation of CEGIS($\mathcal {T}$) are given in Table 2. In combination, CEGIS($\mathcal {T}$)-vbs and CEGIS($\mathcal {T}$)-SMT solve 6 more benchmarks than our straight implementation of CEGIS (42 vs. 36). Unsurprisingly, CEGIS($\mathcal {T}$)-SMT solves more of the invariant generation benchmarks that require synthesising arbitrary constants than CEGIS. Also, CEGIS($\mathcal {T}$)-FM is generally faster than CEGIS for benchmarks with non-trivial constants as it avoids enumerating the constants. However, it fails to solve many of the other benchmarks that plain CEGIS can solve because attempting to apply FM slows it down.

A noted weakness in our implementation of both CEGIS and CEGIS($\mathcal {T}$) is that they are slow to synthesise long expressions. This is due to the iterative-deepening-style search performed by the implementation where, starting with $n=1$, the space of possible programs of size *n* is searched exhaustively before considering those of size $n+1$, and so on.

### CEGIS($\mathcal {T}$) in CVC4

*Benchmarks* We tested CVC4 on the full set of benchmarks across the SyGuS competition^5^ minus the programming-by-example ones. We excluded PBE because there already exists a specific divide and conquer technique for solving these benchmarks [8] that is orthogonal to CEGIS($\mathcal {T}$). We ran two sets of experiments. For the first one, we removed the grammars from all the benchmarks. Notably, this enables CVC4 to use the full grammar from the background theory. In the second experiment, we included only the benchmarks that contain a grammar (e.g. the invariant benchmarks are excluded from this experiment as they don’t contain a grammar). For these benchmarks we removed all constant literals except 0 and 1 and then extended the grammars to permit any constant literal. For example, a syntactic template which originally contains the rules $Int \rightarrow 0$ and $Int \rightarrow 7$ will now contain the rules $Int \rightarrow 0$ and $Int \rightarrow \textit{Any Integer Literal}$. We include 0 and 1 in the grammar so that the synthesis algorithms that do not implement CEGIS($\mathcal {T}$), and instead enumerate through the grammar given are, at least theoretically, capable of solving the benchmarks that need constants.

*Results* In Table 3 we present results comparing five configurations of CVC4: the default behaviour of CVC4 1.7 using CEGIS, CVC4 using CEGIS but adding constant literals from the benchmark to the grammar, the implementation CVC4-CEGIS($\mathcal {T}$), as well as two “virtual best CVC4 solvers”, denoting the fastest results of some of the previous CVC4 configurations.

The configurations presented in Table 3 are as follows:

- CVC4-CEGIS: default behaviour of CVC4 version 1.7 using CEGIS, as described in [34];
- CVC4-ac: CVC4 using CEGIS, but adding constant literals from the benchmark to the grammar;
- CVC4-CEGIS($\mathcal {T}$): CEGIS($\mathcal {T}$) as implemented inside CVC4 and described in Sect. 5.3;
- vbs1: the fastest result of CVC4-CEGIS and CVC4-ac.
- vbs2: the fastest result of CVC4-CEGIS, CVC4-ac and CVC4-CEGIS($\mathcal {T}$).

We exclude from the evaluation the single invocation solver of CVC4, which is not impacted in any way by the CEGIS($\mathcal {T}$) approach. We do not compare against the technique described in Sect. 5.2 because it has been deprecated in CVC4 in favour of the more general one described in Sect. 5.3.

**Table 2:** Prototype CEGIS(T) experimental results – for every set of benchmarks, we give the number of benchmarks solved by each configuration within the timeout and the average time taken per solved benchmark

Benchmark	CEGIS($\mathcal {T}$)-SMT		CEGIS($\mathcal {T}$)-FM		CEGIS		CEGIS-inc		CEGIS($\mathcal {T}$)-vbs	
	#	s	#	s	#	s	#	s	#	s
comparisons	1	0.1	1	0.63	1	0.1	1	0.1	1	0.1
hackers/crypto	3	32.0	3	31.0	3	31.0	3	237.7	3	30.4
inv	23	220.9	14	60.3	17	203.0	17	86.8	23	171.1
other	15	77.7	13	85.1	15	89.2	15	18.7	15	18.6
total solved	42	151.0	31	65.9	36	135.6	36	68.5	42	102.5

**Table 3:** Experimental results for CVC4-CEGIS($\mathcal {T}$) – for every set of benchmarks, we give the number of benchmarks solved by each configuration within the timeout and the average time taken per solved benchmark

Benchmark	Num	CVC4-CEGIS		CVC4-ac		CVC4-CEGIS($\mathcal {T}$)		vbs1(first two)		vbs2(all three)	
		#	s	#	s	#	s	#	s	#	s
With grammar											
Conditional inverses	128	114	0.65	114	0.65	114	25.7	114	0.65	114	0.65
Invertibility conditions	128	94	85.5	94	82.7	101	111.4	94	82.4	102	84.8
General	232	128	40.2	128	40.2	108	81.5	128	40.0	132	31.7
Total (with grammar)	488	336	39.4	336	38.7	323	71.2	336	38.5	348	37.1
Without grammar											
General track (2018)	308	147	98.9	144	57.6	129	152.7	149	65.9	149	60.2
Conditional inverses	161	142	25	142	2.7	144	3.7	142	1.4	144	3.2
Invertibility condition	160	103	84.0	96	84.2	107	83.0	105	71.7	112	66.0
Invariants (woosuk)	369	157	106.3	157	106	157	106	157	105.6	157	105.3
Invariants (lustre)	456	203	84.1	199	126.9	175	95.8	208	106.7	208	106.7
Invariant track (2018)	127	109	4.2	111	8.8	111	29.6	111	4.4	111	3.9
Invariants (code2inv)	276	184	69.2	187	60.4	185	78.7	191	66.4	194	65.7
Total (no grammar)	1808	1042	70.7	1033	68.6	1005	80	1060	65.6	1072	64.1
Total	2296	1378	63.1	1369	61.3	1328	77.9	1396	59.1	1420	57.5

The implementation of CEGIS($\mathcal {T}$) inside CVC4 (as shown in Table 3) is able to extend the set of benchmarks that CVC4 with CEGIS solves by 24. This is shown in the virtual best solver results, where vbs2, including CEGIS($\mathcal {T}$), solves 1420 benchmarks compared to vbs1, which solves 1396. Note that the virtual best solvers are presented not as practical parallel solvers, but to highlight the improvements of CVC4-CEGIS($\mathcal {T}$) w.r.t. CVC4-CEGIS in solving these extra 24 problems. These improvements occur on benchmarks that require large constants, as expected. Moreover, it would be virtually impossible for CVC4-CEGIS to solve the 24 benchmarks that CVC4-CEGIS($\mathcal {T}$) solves exclusively, since representing those large constants in the regular enumeration (which uses only the constants 0 and 1) requires prohibitively large programs. CVC4-CEGIS($\mathcal {T}$) is worse than CVC4-CEGIS on some benchmarks because when no large constants are necessary for building a solution, CEGIS($\mathcal {T}$) may only add overhead.

**Fig. 4:**
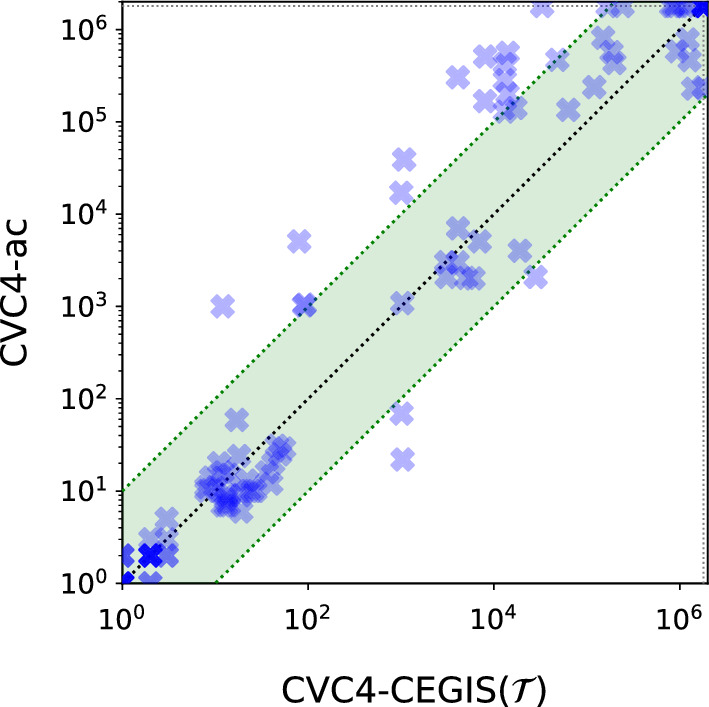
Comparison on the 128 invertibility condition benchmarks with grammars. Execution times in miliseconds

CVC4-CEGIS($\mathcal {T}$) is particularly effective on the invertibility conditions with grammars benchmark set, solving 7 more problems than both CVC4-CEGIS and CVC4-ac (101 benchmarks vs. 94). Moreover it significantly outperforms CVC4-ac on harder benchmarks (those requiring more than 100 s to solve), as shown in Figure 4. These benchmarks contain synthesis goals for inverses of bit-vector operators (for more details, see Niemetz et al. [30]), whose solutions often require particular constants. Even though the grammars in these benchmarks contain carefully chosen constants to help the SyGuS solver [30, Sect 3.1], CVC4-CEGIS($\mathcal {T}$) ability to find arbitrary, necessary, constants still can lead to considerable improvements.

On average, CVC4-CEGIS is better than CVC4-CEGIS($\mathcal {T}$) because on benchmarks where constants are not needed there is some slight overhead to using CEGIS($\mathcal {T}$). However, CEGIS($\mathcal {T}$) is able to solve benchmarks that CEGIS alone cannot and the portfolio of solvers is a winning combination. Also, CVC4-CEGIS performs better than CVC4-ac because providing explicit constants in the grammar requires CVC4 to enumerate through a bigger space. This illustrates the importance of techniques like CEGIS($\mathcal {T}$): it allows finding constants when needed, and avoid enumerating them when they are not needed.

## Related Work

The traditional view of program synthesis is that of synthesis from complete specifications [29]. Such specifications are often unavailable, difficult to write, or expensive to check against using automated verification techniques. This has led to the proposal of inductive synthesis and, more recently, of oracle-based inductive synthesis, in which the complete specification is not available and oracles are queried to choose programs [27].

A well-known application of CEGIS is program sketching [40, 42], where the programmer uses a partial program, called a *sketch*, to describe the desired implementation strategy, and leaves the low-level details of the implementation to an automated synthesis procedure. Inspired by sketching, Syntax-Guided Program Synthesis (SyGuS) [3] requires the user to supplement the logical specification provided to the program synthesiser with a syntactic template that constrains the space of solutions. In contrast to SyGuS, our aim is to improve the efficiency of the exploration to the point that user guidance is no longer required.

Another very active area of program synthesis are component-based approaches [2, 18–20, 23, 24, 32]. Such approaches are concerned with assembling programs from a database of existing components and make use of various techniques, from counterexample-guided synthesis [23] to type-directed search with lightweight SMT-based deduction and partial evaluation [19] and Petri-nets [20]. The techniques developed in the current paper are applicable to any component-based synthesis approach that relies on counterexample-guided inductive synthesis.

Heuristics for constant synthesis are presented in [15], where the solution language is parameterised, inducing a lattice of progressively more expressive languages. One of the parameters is word width, which allows synthesising programs with constants that satisfy the specification for smaller word widths. Subsequently, heuristics extend the program (including the constants) to the required word width. As opposed to this work, CEGIS($\mathcal {T}$) denotes a systematic approach that does not rely on ad-hoc heuristics.

Regarding the use of SMT solvers in program synthesis, they are frequently employed as oracles. By contrast, Reynolds et al. [35] present an efficient encoding able to solve program synthesis constraints directly within an SMT solver. Their approach relies on rephrasing the synthesis constraint as the problem of refuting a universally quantified formula, which can be solved using first-order quantifier instantiation. Conversely, in our approach we maintain a clear separation between the synthesiser and the theory solver, which communicate in a well-defined manner. In Sect. 6, we provide a comprehensive experimental comparison with the synthesiser described in [35].

## Conclusion

We proposed CEGIS($\mathcal {T}$), a new approach to synthesis of programs with non-trivial constants that combines the strengths of a counterexample-guided inductive synthesiser with those of a theory solver with the aim of improving the synthesis of programs with non-trivial constants. We discussed two options for the theory solver, one based on variable elimination and one relying on an off-the-shelf SMT solver for first-order formulas with quantifiers, as well as a case study on integrating CEGIS($\mathcal {T}$) inside CVC4. Our experiments results show a significant performance improvement on benchmarks from the SyGuS competition on benchmarks that require synthesising arbitrary constants.

in this paper, we evaluated CEGIS($\mathcal {T}$) in the context of constant elimination when the secification belongs to linear arithmetic. Other techniques for eliminating existentially quantified variables can be used. For instance, one might use cylindrical algebraic decomposition [12] for specifications with non-linear arithmetic.

## Data Availability

The benchmarks tested during the current work are available at https://github.com/kroening/cegis-smt-journal-paper.git.

## Footnotes

1 Existential second-order logic allows existential quantification over second-order (i.e., function) variables in addition to quantification over (first-order) variables [38].

2 www.github.com/kroening/fastsynth.git.

3 www.github.com/CVC4/CVC4.git.

4 https://docs.aws.amazon.com/awsec2/latest/userguide/compute-optimized-instances.html.

5 https://github.com/SyGuS-Org/benchmarks/tree/master/lib [retrieved March 2021].

## Code Availability

All the code developed and used for the current work is available at www.github.com/kroening/fastsynth.git (the code for fastsynth) and www.github.com/CVC4/CVC4.git (the code for CVC4).
